# In situ simulation-based team training and its significance for transfer of learning to clinical practice—A qualitative focus group interview study of anaesthesia personnel

**DOI:** 10.1186/s12909-023-04201-8

**Published:** 2023-04-04

**Authors:** Anne Strand Finstad, Ingunn Aase, Conrad Arnfinn Bjørshol, Randi Ballangrud

**Affiliations:** 1 Department of Nurse Anaesthesia, Division of Emergencies and Critical Care, Oslo University Hospital, Oslo, Norway; 2 SHARE - Centre for Resilience in Healthcare, Faculty of Health Sciences, University of Stavanger, Stavanger, Norway; 3 The Regional Centre for Emergency Medical Research and Development (RAKOS), Stavanger University Hospital, Stavanger, Norway; 4 Department of Anaesthesiology and Intensive Care, Stavanger University Hospital, Stavanger, Norway; 5 Department of Clinical Medicine, University of Bergen, Bergen, Norway; 6 Department of Health Science, Norwegian University of Science and Technology, Gjøvik, Norway

**Keywords:** Anaesthesia, Interprofessional, In situ simulation-based team training, Non-technical skills, Patient safety

## Abstract

**Background:**

Anaesthesia personnel are an integral part of an interprofessional operating room-team; hence, team-based training in non-technical skills (NTS) are important in preventing adverse events. Quite a few studies have been done on interprofessional in situ simulation-based team training (SBTT). However, research on anaesthesia personnel’s experiences and the significance for transfer of learning to clinical practice is limited. The aim of this study is to explore anaesthesia personnel’s experience from interprofessional in situ SBTT in NTS and its significance for transfer of learning to clinical practice.

**Methods:**

Follow-up focus group interviews with anaesthesia personnel, who had taken part in interprofessional in situ SBTT were conducted. A qualitative inductive content analysis was performed.

**Results:**

Anaesthesia personnel experienced that interprofessional in situ SBTT motivated transfer of learning and provided the opportunity to be aware of own practice regarding NTS and teamwork. One main category, ‘interprofessional in situ SBTT as a contributor to enhance anaesthesia practice’ and three generic categories, ‘interprofessional in situ SBTT motivates learning and improves NTS’, ‘realism in SBTT is important for learning outcome’, and ‘SBTT increases the awareness of teamwork’ illustrated their experiences.

**Conclusions:**

Participants in the interprofessional in situ SBTT gained experiences in coping with emotions and demanding situations, which could be significant for transfer of learning essential for clinical practice. Herein communication and decision-making were highlighted as important learning objectives. Furthermore, participants emphasized the importance of realism and fidelity and debriefing in the learning design.

**Supplementary Information:**

The online version contains supplementary material available at 10.1186/s12909-023-04201-8.

## Background

Adverse events in hospitals are challenging. Adverse events may arise from healthcare teams’ insufficient non-technical skills (NTS*)* [1] and cause intraoperative errors, adverse patient outcomes, and even mortality [2]. Therefore, effective teamwork focusing on NTS is crucial to prevent these occurrences [3]. Integrating patient safety competencies, including NTS pertaining to teamwork and communication in continuing professional development, which emphasize interprofessional learning, could be critical [4]. Simulation-based team training (SBTT) prepares interprofessional teams to successfully manage challenging situations and prevent patient injuries [5].

According to the Healthcare Simulation Dictionary [6] simulation is ‘a technic that creates a situation or environment to allow persons to experience a representation of a real event for the purpose of practice, learning, evaluation, testing, or to gain understanding of systems or human actions’. Furthermore, in situ simulation is ‘taking place in the actual patient care setting/environment in an effort to achieve a high level of fidelity and realism’ [6]. In situ training is particularly suitable for difficult work environments and is valuable to assess, troubleshoot, or develop new system processes [6], and provides a familiar, safe and possibly time effective training [7]. According to Sørensen et al. in situ simulation may also lead to organisational learning [8] where the healthcare personnel put their learning into effect when returning to clinical practice. Kirkpatrick’s four levels of evaluation model can be used to describe the level of learning outcome and is a widely used framework to measure the outcome of SBTT in healthcare [9] The four levels cover: Level 1 – healthcare personnel’s reaction, what they thought and felt about the training; Level 2 – healthcare personnel’s learning, the resulting increase in knowledge or capability; Level 3 – healthcare personnel’s behaviour – extent of behavioural changes in the professional setting, i.e., transfer of learning to the clinical setting; and Level 4 – results – the effect of healthcare professional actions, i.e., improved patient outcomes. Multiple levels are possible within a single study [10].

Human factor focused SBTT is introduced in healthcare by Gaba et al. [11], and anaesthesia personnel were the first to implement this training [12, 13]. ‘Human factors refer to environmental, organisational and job factors, and human and individual characteristics, which influence behaviour at work in a way which can affect health and safety’ [14]. Abildgren et al.’s systematic review [15] refers to several studies finding ‘that adverse events often occurs in non-routine, complex environments due to interactions between humans and the systems in which they work’, and that NTS are a limited part of these aspects [15]. Anaesthesia personnel work with an interprofessional team in the operating room and have a crucial role ensuring patient care and safety e.g. resolving airway complications to prevent adverse events [16–18]. NTS include the cognitive, social and personal resource skills that complement technical skills and contribute to safe and efficient task performance [19]. Flin et al. (2008) describes seven basic NTS important for safe and efficient performance in high-risk settings: situation awareness, decision-making, communication, teamwork, leadership, and the management of stress and fatigue. Among anaesthesia personnel, these skills are regarded as essential for safe clinical practice [17, 20, 21].

Goldshtein et al.’s systematic review [22] reports that in situ SBTT has a positive effect on patient outcomes including reducing mortality and morbidity. Standardised in situ simulation mock codes have increased survival after in-hospital cardiopulmonary arrest [23]. According to Kurup et al., in situ simulation training allows teams to review their own practice and may be cost-effective; however, further assessment of its effectiveness on clinical outcomes is needed [24]. Lorello et al. report inconsistent outcomes after SBTT regarding anaesthesia personnel’s satisfaction, knowledge, skills, and behaviour in a systematic review and meta-analysis [25]. Improved team performance, cultural attitudes, and communication among anaesthesiologists and obstetricians [26], trauma teams [27], and neonatal resuscitation [28] after SBTT are reported. LeBlanc et al. show in a narrative review of literature, the potential role of emotions during simulation-based education. Positive emotions increase cognitive flexibility, while negative emotions decrease the ability to form associations between events. This may be crucial for learning and problem-solving skills [29]. Bearman et al. highlight the usefulness of fallibility as a part of professional practice; hence, self-reflection in simulation-based education provides an opportunity to introspect and learn from failures [30]. Debriefing is a key element in simulation [31], which provides the opportunity to reflect on NTS, such as the importance of ‘speaking up’. Lemke et al. assess experienced anaesthesia personnel’s speaking up behaviours and its consequent reactions during anaesthesia induction and describe on the complexity of speaking up and its importance for patient safety [32].

However, exploring the transfer of learning from simulation to clinical practice remains uncertain [33]. A recent systematic review concludes that research on the retention and transfer of human factor skills from SBTT to clinical practice is insufficient and further research is essential to gain knowledge of its effect on patient safety [15]. In our study we primarily focus on the application of learning captured by Kirkpatrick’s Level 1, what the anaesthesia personnel’s thought and felt about the SBTT, e.g. which emphasizes it’s relevance for their clinical practice, Level 2, what they describe as increase in knowledge or intellectual capability from before to after the training, and Level 3, transfer of learning to clinical practice.

To our knowledge, there are no studies on anaesthesia personnel’s experience of interprofessional in situ SBTT of NTS and its significance for transfer of learning to clinical practice. This qualitative study based on follow-up interviews during a six-month period will provide in-depth knowledge which might contribute to strengthen the anaesthesia personnel continuing improvement with a goal for professional development and a safer practice.

The aim of this study is to explore anaesthesia personnel’s experience from interprofessional in situ SBTT in NTS and its significance for transfer of learning to clinical practice.

The research questions are:

1. How do nurse anaesthetists and anaesthesiologists experience the in situ SBTT in NTS two weeks and six months after the training?
2. How do nurse anaesthetists and anaesthesiologists experience the significance for transfer of learning of NTS to clinical practice two weeks and six months after the training?

## Methods

### Design

This qualitative descriptive study design [34] was based on focus group interviews, two weeks and six months after SBTT. Using focus groups give the opportunity to acquire viewpoints of several respondents in a short period of time. We expected the method, emphasizing group interaction and discussions, to give us rich and deep expressions of various experiences, opinions, and informative data [35].

### Setting and sample

In a Norwegian university hospital’s surgical department with 60 nurse anaesthetists and 22 anaesthesiologists employed, performing emergency caesarean sections as well as other operations, an interprofessional in situ SBTT was ongoing. Our study included five training sessions implemented during 17 weeks throughout the autumn of 2018 where a total of 14 anaesthesia personnel (ten nurse anaesthetists and four anaesthesiologists) participated. Anaesthesia personnel who participated were asked to attend focus group interviews two weeks after the training (interview 1) to get their experience close to the SBTT, and six months later (interview 2) to get their experience after having returned to clinical practice for a while. All fourteen anaesthesia personnel accepted to participate. Participants’ characteristics (gender, age, and years of experience with SBTT) are presented in Table 1.

**Table 1:** Characteristics of the participants (*n* = 14)

Background	Subgroup	Mean (median)	N (%)
Professions	Nurse anaesthetist		10 (71.4)
	Anaesthesiologist		4 (28.6)
Age			
	29–39 years	44.5 (43)	5 (35.7)
	40–49 years		4 (28.6)
	50–61 years		5 (35.7)
Gender	Female		7 (50)
	Male		7 (50)
Prior experience with SBTT	Yes		14 (100.0)
	1–2 times		3
	3–5 times		5
	5–10 times		4
	> 10 times		2

### The interprofessional in situ SBTT programme

An interprofessional in situ SBTT programme was developed via the collaboration between the obstetrics, anaesthesia, and surgical departments in a university hospital. The SBTT planning group comprised representatives from each profession and an educated facilitator (ASF) for the pedagogical aspect [36]. The SBTT programme was based on the Simulation Setting Model by Peter Dieckmann [37, 38]. The model contains seven prototypical phases that can be modified in order and number according to the actual training programme [38] (Table 2 presents the modified model used in this study).

**Table 2:** The present study’s simulation-based team training programme phases. Adapted from Dieckmann, P. [37]

1	Introduction Theory Inputs	The participants received an information sheet before the SBTT with theory inputs regarding medical simulation and NTS
2	Simulation and Scenario Briefing	The participants received information about the simulation environment, equipment, simulated patient safety, confidentiality, learning objectives, and the scenario
3	Scenario – Simulation Number 1	The participants were enacting a scenario case, which formed the basis of the first debriefing
4	Debriefing	The participants attended a structured professional and interprofessional discussion of the scenario actions
5	Scenario – Simulation Number 2	The participants were enacting the scenario case for a second time, which formed the basis of the second debriefing and evaluation
6	Debriefing	The participants attended a structured professional and interprofessional discussion of the scenario actions for a second time
7	Ending/Evaluation	The participants took part in a summary and evaluation session of their satisfaction with the SBTT

The duration of the interprofessional in situ SBTT programme was 17 weeks during the autumn of 2018. The on-duty surgical team (nine professionals: two obstetricians, one midwife, one paediatric nurse, two operation nurses, two nurse anaesthetists, and one anaesthesiologist) were recruited to the SBTT, and the scenario was an emergency caesarean section (Table 3 describes the scenario in detail).

**Table 3:** In situ SBTT programme’s learning objectives and simulation scenario

SBTT learning objectives	• Shared responsibilities
	• Communicate clearly and concisely
	• Awareness of the situation
	• Make effective reports
	• Achieve acceptable response time
	• Performing correct medical treatment
SBTT scenario	A 36-year-old in first-time pregnancy close to term arrived at the maternity ward. Normal pregnancy, except gestational diabetes, control a week ago showed the foetus at the 90th percentile. The mother’s body mass index (BMI) was 40 at start of pregnancy. Labour proceeding normally. Normal Cardiotocography (CTG). Continuous monitoring. Epidural labour anaesthesia. Oxytocin infusion. No progression during the last 30 min. Now foetal bradycardia, (pulse 80). On-call obstetrician is notified. Emergency caesarean section calling is activated. During transport from the maternity ward to the operation room, the mother is anxious, crying, in pain, and has tachycardia and high blood pressure

A midwife acted as the simulated patient. In the introduction phase, a few days before the SBTT, the participants were provided an information sheet regarding medical simulation and NTS (Table 2). The SBBT sessions (each lasting one hour) (Tables 2, 3) were conducted according with the department’s schedule. The briefing session included presentation of the facilitator and the observers (one representative from each profession in the planning group). The participants’ previous experience with simulation training were registered, and information according to in situ environments in the operating room, e.g. equipment, simulated patient, fidelity, participants’ opportunity, were given. Learning objectives, based on experiences done by the planning group, were presented, with an opportunity to ask questions. The learning objectives covering the needs for anaesthesia personnel as key team members in an interprofessional surgical team in ensuring patient care and safety (Table 3 describes the chosen learning objectives). The scenarios were performed with the facilitator (ASF) and observers discretely positioned in the operation room. The facilitator (ASF) conducted debriefing including descriptive, analytic and reflective phases [31] and the observers provided feedback and professional support. A second debriefing ended with a summary and evaluation (Tables 2, 3).

### Data collection

A semi-structured interview guide based on open-ended questions (Additional file 1) was used. The questions were specifically designed to gain knowledge on the various facets of the interprofessional in situ SBTT and provided the participants with the opportunity to holistically comprehend its advantage in clinical practice [39]. The interview guide was validated via a pilot interview and no changes were made. The open-ended questions pertained to the anaesthesia personnel’s experiences of SBTT and transfer of learning to clinical practice, including usefulness, transferability, outcome, implementation, challenge, and benefit (see interview guide in Additional file 1).

Data were collected via focus group interviews two weeks (interview 1), and six months (interview 2) after the SBTT programme during the period from September 2018 to November 2019. The benefits for generating data at two time points, was to get a longitudinal perspective of knowledge sustainability of transfer of learning to clinical practice. Five focus groups consisted of both professions (nurse anaesthetist and anaesthesiologist) and two focus groups consisted of one profession (nurse anaesthetists) with two to five participants in each group. The participants were allocated to focus groups according to their clinical shifts and availability. A total of 14 anaesthesia personnel participated in interview 1 (four focus groups) and a total of 11 anaesthesia personnel participated in interview 2 (three focus groups), the remaining participants were not available. Due to time available and clinical shifts, new constellations were inevitable. The anaesthesia personnel in interview 2 also participated in interview 1 (Table 4).

**Table 4:** Participants in the focus groups

Interview 1: 2 weeks after SBTT (September 2018)		
Focus group	Nurse Anaesthetist (n)	Anaesthesiologist (n)
1 (*n* = 3)	2	1
2 (*n* = 3)	1	2
3 (*n* = 3)	3	0
4 (*n* = 5)	4	1
**Interview 2: 6 months after SBTT (February 2019)**		
Focus group	Nurse Anaesthetist (n)	Anaesthesiologist (n)
5 (*n* = 4)	2	2
6 (*n* = 2)	2	0
7 (*n* = 5)	4	1

The interview duration was approximately one hour. The moderator (ASF) and observer (RB), who made field notes, conducted all the interviews. The moderator (ASF) presented an introduction of the study and led the discussions. A summary of data from the interview was read aloud by the observer (RB) and was confirmed by the participants in each focus group. The interviews were audio-recorded, transcribed verbatim, and anonymised before analysis by the moderator. Data saturation was assessed to be sufficient according to information power [40].

### Data analyses

A qualitative manifest and inductive content analysis, based on Elo and Kyngäs’ method [41], was used to gain insights into anaesthesia personnel’s experience from interprofessional in situ SBTT in NTS and transfer of learning to clinical practice.

The analysis was structured into three phases: preparing, organising, and reporting [41]. In the preparing phase, the first author (ASF) transcribed the interviews and all authors (ASF, IA, CAB, and RB) read the interviews several times to gain familiarity with the text and understand the content of the participants’ statements [41]; subsequently, the interviews were individually analysed. In the organising phase, all the authors participated throughout the analysis process to identify codes. Data was split into smaller data extract and labelled with a code which seemed to be relevant and meaningful considering the study aim. Based on similarities and differences, the codes were sorted into sub-categories, which were interpreted and, finally, aggregated into broader generic categories and finally a main category, after discussions among the authors.

The analysis generated one main and three generic categories, and seven sub-categories.

In the reporting phase, an overview of the abstraction process with the generation of categories was made (Table 5) and the results were described using the content of the sub-categories. The authors agreed on the citations to supplement the text.

**Table 5:** An overview of generation of categories

Sub-category	Generic category	Main category
• Provides the team an experience of coping	➢ Interprofessional in situ SBTT motivates learning and improves NTS	Interprofessional in situ SBTT as a contributor to enhance anaesthesia practice
• Enables improvement of NTS for clinical practice		
• Facilitates informative professional and interprofessional discussions		
• Provides the opportunity to be aware of own practice	➢ Realism in SBTT is important for learning outcome	
• Use of a simulated patient may increase or decrease realism		
• Helps clarify the roles in the interprofessional team	➢ SBTT increases the awareness of teamwork	
• Precise communication contributes to clarity		

The analysis was performed in the original Norwegian language and the authors approved the translation. The results are reported according to the COREQ Checklist [42] (Additional file 2).

## Results

The main category ‘Interprofessional in situ SBTT as a contributor to enhance anaesthesia practice’ describes anaesthesia personnel’s experience of the SBTT and its significance for transfer of learning to improve NTS in clinical practice. The selected quotes are used to illustrate results [43]. The main category was generated from three generic and seven sub-categories (Table 5).

### Interprofessional in situ SBTT motivates learning and improves NTS

This generic category pertains to the participants’ experience of the in situ SBTT as a facilitator for coping, learning, and improvement, with a view to clinical practice. This category has three sub-categories: ‘provides the team an experience of coping’, ‘enables improvement of NTS for clinical practice’, and ‘facilitates informative professional and interprofessional discussions’.

#### Provides the team an experience of coping

The participants described in situ SBTT as a programme facilitating learning and training, including detecting failure. With regard to significance for transfer of learning, they stated that diverted attention affected situation awareness. Greater concentration on their own tasks, such as electronic documentation, affected the attention regarding the patient and surgical team.

Uncertainty and demanding technical skills were challenges that resulted in stress, ‘an unsteady hand’, and ‘weird actions’ (mental process) and was reported as important elements for simulation training. Mumbling and confusion in the first scenario were interpreted as uncertainty.

*When you are unsure of the situation, then you become reserved. (No. 2.2).*

Some participants disliked being observed, and not performing as expected, led to negative emotions. After six months, some participants did not suppress the disappointment resulting from a bad performance in the first scenario. They suggested that the fear of failure and the ‘feeling of being tested’ influenced their performance; however, the second scenario was an opportunity to correct earlier mistakes and provided an experience of coping. Some expressed after two weeks:

*Without the second scenario, I would probably gone home with a bad feeling of not coping with this teamwork in a real situation... (2.3) It is important to get the feeling of team coping. (2.2)*

The participants perceived the training as informative, and the learning was prominent after their mistakes.

#### Enables improvement of NTS for clinical practice

Most of the participants had the opinion that the SBTT was positive, instructive, and useful with regard to significance for transfer of learning to similar emergencies in clinical practice. As one said after two weeks:

*It is better to make mistakes during the simulation training, better to be watched when almost doing something wrong, than actually doing it three weeks later (in clinical practice). (No.3.1).*

Six months later, some participants experienced transfer of learning according to better NTS in clinical practice, e.g. get acquainted with the surgical team, while others stated that the knowledge had declined. Frequency and participation from everyone (in the team) were considered to be the keys to success. One expressed:

*If it takes a year before next simulation training, then I can’t say it has any effect on my behaviour in clinical practice (No.7.3) … it is fresh produce (No.5.3).*

When some participants experienced transferred learning of NTS in clinical practice, it inspired others to adopt them, e.g., closed loop communication.

The participants suggested including more theoretical knowledge prior to SBTT to be better prepared for learning. The scenario, an emergency caesarean section, was considered emotionally dramatic, which may improve recall. However, one participant said:

*The more complex the scenario is, the more you focus on your own team [anaesthesia team]. (No.4.5)*

The participants stated that in a simpler scenario, it could be easier to open up, communicate, and observe everyone in the surgical team.

#### Facilitates informative professional and interprofessional discussions

The participants stated that debriefing provides the opportunity to discuss the scenario-case performance and speak up in a structured way.

They suggested allocating more time to reflect on details, which would increase the learning outcome. They had less time during the second debriefing because they had to return to work immediately. After two weeks, one participant said:

*The most relevant discussion is here [in the interview] … (No.4.2)*

In clinical practice, debriefing was usually related to serious cases and personnel had an opportunity to reflect on their performance. They emphasised that debriefing with interprofessional discussions, both in SBTT and as significance for transfer of learning to clinical practice, improved their behaviour. There was a need for the anaesthesia team to have a short debriefing according to their specific tasks, with regard to transfer of learning. Some participants considered this to be of no interest for the others, while others disagreed. One said:

*To find the key to good collaboration in team, you have to know what is important for the other professions in your team. (No.4.4)*

### Realism in SBTT is important for learning outcome

In a longitudinal perspective (two weeks and six months after SBTT), this generic category pertains to the importance of realism in SBTT to provoke emotions, manage stressful situations, disclose practical challenges, and conduct patient treatment, with a view to transfer of learning to clinical practice.

This category has two sub-categories: ‘‘provides the opportunity to be aware of own practice’’ and ‘‘use of a simulated patient may increase or decrease realism’’.

#### Provides the opportunity to be aware of own practice

The participants experienced interprofessional in situ SBTT as an opportunity to reflect on and change their own clinical practice, such as replacing equipment and managing stress. Participants were aware of emotions in the scenarios, and after two weeks and six months respectively, they explained:

*…simulation can initiate so many physical and psychological processes in the body, like actually being there. (No.1.2) In a successful simulation scenario, you can feel an increased pulse rate… (No. 7.1)*

Participants suggested a need for realism and frequency to obtain a type of muscle memory, which could release more energy for mental work in clinical practice. Some reported that the SBTT situation was similar to an earlier clinical experience, for example time pressure. They meant that it was significant for transfer of learning in managing frustration and stress in an interprofessional team in clinical practice.

#### Use of a simulated patient may increase or decrease realism

The participants appreciated a simulated patient; however, some found visualising a full-term pregnant patient with physical problems difficult, when the simulated patient was ‘small and thin’.

As two participants said:

*It was a bit disturbing with a simulated patient with a pillow on her stomach – you are not in real life anymore (No. 2.3) …forgot that it was an obese patient (No. 7.3).*

They could lose the ‘feeling of thinking twice’ before inducing general anaesthesia. Others said that SBTT requires imagination to some extent, and it depends on the ability to visualise.

On the other hand, one said:

*In simulation you easily focus on what to do, to do a good job, …perhaps mainly on technical issues, not to make mistakes, …so you can end up with the opposite, that you forget the patient because you are so occupied with what to do… (No. 6.2).*

A short break (‘time-out’) to clarify misunderstandings was conducted during the SBTT and was considered as significant for transfer of learning to be applied in clinical practice.

According to the participants, the simulated patient perceived the anaesthesia personnel’s treatment as rougher when the situation became more intense and serious; however, she felt safer, calmer, and more cared for in the second scenario. The participants considered this as significant for transfer of learning, to clinical practice.

### SBTT increases the awareness of teamwork

This generic category pertains to the professions and their roles in the surgical team, and the positive and challenging communication situation, regarding transfer of learning to clinical teamwork.

This category has two sub-categories: ‘helps clarify the roles in the interprofessional team’ and ‘precise communication contributes to clarity’.

#### Helps clarify the roles in the interprofessional team

After two weeks and six months the participants reflected on the interprofessional team and emphasized that the SBTT learning was transferred to different clinical team settings. Awareness of each person’s role and action in SBTT was experienced as significant for transfer of learning and made teamwork easier in clinical practice. The surgical team was described as containing three smaller teams: the anaesthesia, gynaecologic, and operating nurse teams. Though the surgical team leader was the gynaecologist, this seemed to be unclear for most participants ahead of the training. The participants stated that it was easier to be attentive and act when the team leader was identified, and he or she spoke ‘loud and clear’. The team leader was expected to comprehend the situation and encourage good communication. Therefore, teamwork apparently depended on the persons in the team. An example:

*In the scenario, the midwife took some space, and she was the one who communicated with the patient … and gave good instructions too, then I thought she should be allowed to keep on doing that and not being interrupted by another one (me) … (No. 2.2).*

Despite having different personnel in anaesthesia teams, both in clinical practice and SBTT, the participants experienced a similarity in the team situation and a feeling of safety. During combined training programmes, depending on the frequency, they stated that they become better acquainted, with a view to transfer of learning. Depending on the learning objectives, the participants suggested separate training for the anaesthesia team, even though the interprofessional teamwork was crucial regarding clinical practice.

#### Precise communication contributes to clarity

The participants emphasized teamwork communication and the necessity of the team leader to ‘think aloud’ to enable the team members to plan and execute their own actions and provide feedback. The participants reported disagreements between team members’ opinion of good or bad communication; precise communication could have solved a critical situation that occurred in the SBTT. Several participants experienced improved communications in the second scenario, which could be crucial for transfer of learning to clinical settings. One anaesthesiologist said:

*In the second scenario, the gynaecologist on-call gave specific messages to me and understood the point, so that helped a lot. (No. 2.1).*

The participants described that the noise in the simulated operating room varied from highly disturbing to low and inaudible, which could be essential for nervous patients. In an unsettled surgical team situation (with a lot of noise) in the clinic, for example when an alarm was aroused, the nurse anaesthetist could choose to pay attention to only the anaesthesiologist. The need for time-out to clarify misunderstandings in SBTT was highlighted as significant for transfer of learning. The participants perceived the second scenario as less noisy, and they focused more on ‘whom and what to listen to’ and ‘which messages to give’. The team leader took more control and spoke clearly. The participants described the communication within the anaesthesia team as good, and they said respectively after two weeks and six months:

*When there are messages, about what to do, then you get things done, which could relieve some available time for communication with the patient to calm down and increase safety. (No. 2.2) The use of closed loop communication improves awareness and is a good recall for information already present. (No. 5.3)*

Being aware of the roles and how the communication went on, was experienced as significant for transfer of learning to clinical practice.

## Discussion

We aimed to explore anaesthesia personnel’s experience from interprofessional in situ SBTT in NTS and its significance for transfer of learning to clinical practice. The exploration was conducted in a longitudinal perspective (two weeks and six months after SBTT) to achieve information richness [40]. The results revealed interprofessional in situ SBTT as a contributor to enhance anaesthesia practice. SBTT creates motivation for learning and improvement of NTS, where realism is important for learning outcome and SBTT also increases the awareness of teamwork. The point of time for interviews is mentioned when relevant.

### Interprofessional in situ SBTT motivates learning and improves NTS

The anaesthesia personnel who participated in the study experienced the dramatic scenario similar to cases in actual clinical practice with emotional involvement; thus, it was easy to remember, and hence highly relevant for transfer of learning. Emergency-based scenarios are relevant for SBTT [3], in this context interprofessional in situ SBTT. Le Blanc et al. proclaimed though, that knowledge of a situation may be inferior in high stress rather than in low stress scenarios [29]. The participants in our study stated that a less dramatic scenario could have focused their attention more on NTS, such as communication and teamwork, with the opportunity for transfer of learning. However, simulating a less dramatic emergency caesarean section, which is in reality dramatic, is challenging. Being observed was unpleasant for the participants, although a recent study reported observation, both in actual situations and during in situ training, as a useful way of learning; this was dependent on the facilitator’s skills [7]. Emotions are essential in decision-making [29, 44]. The participants described a reserved behaviour and ‘an unsteady hand’ in unsure clinical situations, in accordance with the influence of negative emotions on situation awareness [29]. Some of the participants mentioned the disappointment, the negative emotion, even after six months. This could happen after clinical experiences as well, which indicates the need for debriefings both in SBTT and clinical practice [45]. Planned and announced simulations, and well-known safe environments, like in our study, could be crucial for learning [7, 8, 25, 46, 47]; hence, this SBTT programme focused on managing challenging clinical situations.

The first scenario performance had resulted in a feeling of failure; however, the first debriefing and second scenario (similar to the first scenario) afforded an opportunity to get a sense of coping, which was confirmed by the participants, in line with Bearman et al.’s ‘learning from self-reflection’, and Jirativanont et al.’s ‘self-assessment of NTS [30, 48]. This experience of coping could be significant for transfer of learning in clinical practice, e.g. in decision-making, where, as mentioned, emotions are essential [29]. A randomised controlled trial has suggested that stress management training improves performance in demanding situations and reduces long-term stress-related effects, such as sleep disorders and burnout [49].The participants requested realistic and frequent SBTT to obtain muscle memory [50]; this may provide more energy for mental work and provide an opportunity for improving NTS, which in turn would be advantageous in demanding situations such as emergencies in clinical practice. This requires frequent in situ SBTT, which may be a challenge for management to prioritize. SBTT reduces stress and promotes team coping strategies, which is beneficial in clinical practice for the personnel, and contributes to patient safety [49]. Implementing this knowledge in clinical practice may be challenging [51]; however, the participants encountered colleagues who inspired others with NTS and this could be a contribution to implementation.

The participants indicated that the post-SBTT discussion (in the interviews) was most relevant, confirming a need for increasing the SBTT debriefing time. Debriefing, an essential element in simulation, facilitates informative professional and interprofessional discussions [31], and according to the participants, it provides the opportunity to speak up, and may disclose crucial human factors [14]. Although lack of time was reported, debriefing is a key to successful SBTT [52]. Time constraints in clinical practice could be a challenge for the facilitator since each phase is time-consuming. Timing and planning are key factors in implementing in situ SBTT [51], and the participants emphasized the presence of an educated facilitator who could use his or her competence for time management. Although there was a need for more internal discussions within the anaesthesia team, the participants highlighted the necessity of interprofessional insight to improve the teamwork, in line with Gittell et al.’s ‘improving healthcare through relationships’ [53]. The one-hour duration of the SBTT may not be adequate to accommodate both interprofessional and anaesthesia team and a follow-up debriefing immediately after SBTT may be required for optimal transfer of learning to clinical practice. The participants considered debriefing both in in situ SBTT and clinical practice as essential. Debriefing in SBTT, could be used as training for debriefing in actual clinical practice and encourage its use in the clinical practice. It requires an observant department manager and an educated facilitator [51], among others, and can lead to system improvements [54].

What the anaesthesia personnel thought and felt about the relevance of in situ SBTT itself and their experiences of the possibility for transfer of learning, may be linked to their experience of learning at Kirkpatrick levels one, two and three [10].

### Realism in SBTT is important for learning outcome

In situ simulation provides realism and the opportunity to be aware of one’s own practice. The participants experienced physical and psychological changes during in situ training. Sørensen et al. suggest that the choice of setting for simulations does not seem to influence individual and team learning, but that in situ simulation could gain organisational learning [8]. This could reveal workplace-specific challenges, such as equipment, guidelines, culture, and communication systems or something unexpected; this is known as system probing. Awareness of these challenges could be crucial for further SBTT and significant for transfer of learning to clinical practice [55, 56]. Emotions may be an advantage or a disadvantage, as mentioned; therefore, being aware of emotional effects and opportunities could be a valuable contribution to the culture of learning. Emphasising psychological safety could result in more open and honest communication and prevent failures in teamwork and potential threats to patient safety in clinical practice [57].

The participants welcomed a simulated patient; the midwife, who displayed great sensitivity to the situation. However, her body mass index was not comparable to the simulated patient’s body mass index, which was an important factor in the scenario aimed at managing the demanding situation. They (participants) also claimed that visualization is important in simulation training and depends on the participant’s imagination to some extent. High-fidelity simulations could enhance learning outcome, but little is known about the correlation between fidelity and learning [58]. According to Sørensen et al. the semantic and motivational context could be more important than physical fidelity [8]. Nevertheless, the participants reported fidelity as a crucial prerequisite for simulation-based problem-solving and learning outcome, regarding significance for transfer of learning to clinical practice.

Training in a realistic and safe environment, a characteristic of SBTT [25], was appreciated by the participants. Adverse events have occurred during in situ simulation training; hence, there is a need to develop a simulation safety policy. The difference between simulated and actual practice could sometimes be unclear [59]. According to the participants, the simulated patient in our study reported less cordial treatment when the situation became intense. This could both be a case for debrief discussion and significant for transfer of learning to clinical practice; however, it was an unpleasant experience for the simulated patient. The participants’ sensitivity was sometimes overwhelming and may have harmed the simulated patient. An educated facilitator is required for enforcing strict guidelines, including emergency call systems, orientation to the environment, training equipment, and simulated patient care.

The participants experienced realism as significant for transfer of learning, which is in line with Kirkpatrick’s level one and two [10].

### SBTT increases the awareness of teamwork

In situ SBTT could help in role clarification in the interprofessional team, as confirmed by the study participants. They became better acquainted during briefing, scenario, and debriefing, [38] resulting in an improved awareness, and they complemented one another in the team. This could be significant for transfer of learning to clinical practice. A shared understanding of each other’s role could decrease the risk of making errors [8]. In the present study, the surgical team leader provided an opportunity for the team members to be more attentive and they responded when he or she spoke up. Precise communication and speaking up, which depended on the team members, was highlighted as a contribution to the clarity of team structure, and significant for transfer of learning to daily clinical practice. Lemke et al. describes the complexity of speaking up, e.g. the risk of unwanted answers and oblique hints [32]. This could be a reason why one team leader in our study did not speak up. A positive example from the in situ SBTT`s scenario – simulation number 2, where they were enacting the scenario case for a second time (Table 2), was the anaesthesiologist who received a specific message from the gynaecologist (team leader), which resulted in better communication. In this way the second scenario gave an opportunity for correction of potential misunderstandings, a risk factor for undesired events. This resulted in improved NTS among the team members and could be significant for transfer of learning to clinical practice.

The participants’ experiences with increased awareness of teamwork may indicate increase in knowledge and capability, in line with Kirkpatrick’s level two of learning outcome [10].

This study has some limitations, which may have influenced the results. Although the entire surgical team participated in the in situ SBTT, only the anaesthesia personnel were asked to participate in focus group interviews. Due to time available and clinical shifts, not every anaesthesia personnel who took part in interview 1 participated in interview 2. One focus group had two participants, three groups had three participants and remainder had four to five participants, and the limited number of participants in the focus group size may have influenced the results. Although a small group also provide important discussions related to the topic. The literature recommends 4–12 informants, but claims that quality of the data is more important than the quantity. Every voice counts, and small groups could be more comfortable according to speaking up, but it depends on the moderator (interviewer), who plays a critical role for the success [35, 40, 41, 60, 61].

Two professions participated in the same focus groups, which could impact the results due to e.g. hierarchical ranking. The fact that the first author is a nurse anaesthetist and both facilitated the in situ simulation and conducted the focus group interviews may have influenced the results.

## Conclusion

Anaesthesia personnel gained considerable experience from the interprofessional in situ SBTT in NTS, which could be significant for transfer of learning essential for clinical practice. This included the role of emotions in coping with demanding situations, importance of good communication within the interprofessional team, including a defined team leader, the importance of realism and fidelity, which is crucial for decision-making. In conjunction with interprofessional discussions in SBTT debriefing and professional discussions in interviews two weeks and six months after SBTT, this led to awareness of their own clinical practice, with possible significance for transfer of learning. However, the participants requested a higher frequency of SBTT and more debriefing time both in SBTT and in clinical practice. The study’s results, which seem to be in line with level one, two and three in Kirkpatrick’s evaluation model [10], may help in improving the organisation of in situ SBTT, draw attention to significance for transfer of learning to clinical practice and contribute to avoiding adverse events and prevent patient injuries.

Further research with observing and rating of anaesthesia personnel’s’ NTS in clinical practice before and after SBTT could be a valuable contribution to study transfer of learning to clinical practice. Further research is needed to explore the entire surgical team’s experience to gain a broader perspective of in situ SBTT, and to what degree it transfers into clinical practice.

## Authors’ information

ASF – Anne Strand Finstad, MD, PhD candidate, Nurse Anaesthetist, Educated Facilitator^1,2^; IA – Ingunn Aase, PhD, Professor^2^; CAB – Conrad Arnfinn Bjørshol, PhD, Anaesthesiology Consultant, Associate Professor^3,^^4,5^; RB – Randi Ballangrud, PhD, Associate Professor, RNT, RN^6^.

## Supplementary Information

**Additional file 1.** Interview guide.

**Additional file 2.** COREQ(COnsolidated criteria for REporting Qualitative research) Checklist.

## Data Availability

The study data and material cannot be made publicly available due to the participants’ consent agreement and the confidentiality policy. Parts of the dataset (in Norwegian language) can be made available upon request to the corresponding author if the local Institutional Data Protection Office of the respective hospital permits.

## Abbreviations

NTS: Non-technical skills
SBTT: Simulation-based team training
