# Supplementary material for: In situ simulation-based team training and its significance for transfer of learning to clinical practice—A qualitative focus group interview study of anaesthesia personnel
Source: BMC Med Educ. 2023 Apr 4;23:208. doi: 10.1186/s12909-023-04201-8 (PMC10071610; doi:10.1186/s12909-023-04201-8)
Supplement: Supplementary file 1 — Additional file 1. Interview guide. [file 12909_2023_4201_MOESM1_ESM.pdf]

Additional file 1: Interview guide

Focus group interview 2 weeks after SBTT

Participants from same type of SBTT; same information, briefing, learning objectives, and debriefing.

Introduction:	Information to participants about the project:<br>- aim<br>- study 2<br>- research question study 2<br>- focus group interview as a method<br>- anonymity and confidentiality
Preliminary questions:	The participants wrote the first three answers individually before the interview started, to save time
	Position, age, and gender
	How long has it been since you participated in SBTT
	How many times have you participated in SBTT?
Open ended questions:	
	How was it to participate in SBTT?
	Was it useful with a view to practice in the clinic?
	Have you transferred some knowledge from SBTT to clinical practice?
	Have you already experienced any outcome?
	If yes, how does it work?
	If no, why does it not work?
	What are the requirements to implement knowledge to the clinical practice?
	Example of situations where knowledge has been transferred to clinical practice?
	What is challenging in clinical practice?
	Examples of difficult situations according to the use of knowledge?
	Is it possible to practice this in simulation training?
	How do you think this knowledge could benefit the patient?

Focus group interview 6 months after SBTT

Participants from focus group interviews 2 weeks after SBTT.

Introduction	Repeat information to participants about the project:<br>- aim<br>- study 2<br>- research question study 2<br>- focus group interview as a method<br>- anonymity and confidentiality
Preliminary questions:	The participants wrote the first three answers individually before the interview started, to save time
	Position, age, and gender
	How long has it been since you participated in SBTT, emergency caesarean section scenario?
	How many times have you participated in other SBTT after the SBTT, emergency caesarean section scenario?
Open ended questions:	
	Have you transferred any knowledge relating to non-technical skills from the SBTT to clinical practice?
	If yes, what are your experiences in clinical practice?
	If no, are there any challenges?
	What does it take to implement knowledge relating to non-technical skills in clinical practice?
	Can you give any example from situations and the use of non-technical skills?
	What could be a challenge? (e.g., culture, hierarchy, and domination technique)
	Examples of difficult situations according to the use of non-technical skills?
	Do you think it is possible to practice this in SBTT? Any suggestions?
	How do you think your knowledge relating to non-technical skills can be useful for the patient?
